# Assessing Quality, Readability, and Transparency of Online and AI-Generated Information on Type 2 Diabetes Mellitus: Cross-Sectional Exploratory Study

**DOI:** 10.2196/93822

**Published:** 2026-07-15

**Authors:** Sripradha Srinivasan, Ravanth Baskaran, Srinjay Mukhopadhyay, Mayank Patel

**Affiliations:** 1 University Hospital Southampton NHS Foundation Trust, Southampton, England, United Kingdom; 2 School of Medicine, Cardiff University, Cardiff, Wales, United Kingdom; 3 National Heart and Lung Institute, Imperial College London, London, United Kingdom; 4 Imperial College Healthcare NHS Trust, The Bays, S Wharf Rd, London, England, W2 1NY, United Kingdom, 44 02033113311

**Keywords:** artificial intelligence, AI, ChatGPT, digital health, online patient information, type 2 diabetes mellitus

## Abstract

**Background:**

Type 2 diabetes mellitus (T2DM) affects approximately 590 million people worldwide, and its management relies heavily on patient education. With the emergence of online health information and artificial intelligence (AI) large language models, patients are increasingly sourcing medical information independently.

**Objective:**

This study compared the quality, readability, and transparency of websites and AI-generated leaflets (AIGLs) related to T2DM.

**Methods:**

Four predefined search terms (“type 2 diabetes,” “type 2 diabetes mellitus,” “T2DM,” and “adult diabetes”) were entered into 3 major search engines (Google, Yahoo, and Bing), and the top 20 search results were retrieved. AIGLs with patient information about T2DM were produced using a standardized prompt in 4 AI large language models (ChatGPT, Gemini, DeepSeek, and Grok). Information quality was assessed using the DISCERN score, calculated by 3 independent raters and ChatGPT. The *Journal of the American Medical Association (JAMA)* benchmarks were used to measure reliability and transparency. The Flesch-Kincaid Grade Level was used to determine readability.

**Results:**

Seventy-five websites and 4 AIGLs were evaluated. Mean author-rated DISCERN scores were 42.6 (SD 11.3) for websites and 43.9 (SD 1.74) for AIGLs, corresponding to fair quality (DISCERN 41‐51). In contrast, ChatGPT-rated mean DISCERN scores were higher, with 58.5 (SD 11.5) for websites and 61.0 (SD 2.94) for AIGLs, corresponding to good quality (DISCERN 52‐63). Mean *JAMA* benchmark scores were 2.74 (SD 0.965) for websites, indicating moderate reliability (2-3 out of 4 points), whereas all AIGLs scored 0 out of 4 points. Mean Flesch-Kincaid Grade Level scores for websites were 8.67 (SD 2.23) and 8.30 (SD 1.92) for AIGLs, corresponding to an eighth- to ninth-grade comprehension level. Spearman rank correlation demonstrated minimal variability among the 3 independent raters but showed a significant difference between ChatGPT and the 3 independent raters.

**Conclusions:**

Given the high prevalence of T2DM, both websites and AIGLs demonstrated suboptimal quality, readability, and transparency. Increasing patient reliance on digital health information calls for improved readability standards and stronger safeguards for AI-generated content. Both websites and AIGLs require an eighth- to ninth-grade comprehension level, far above the average reading age of 9 years in the United Kingdom (fourth- to fifth-grade level). This reduces the accessibility of online health information. The landscape of medical consultations is evolving, with patients increasingly presenting with preconceived notions based on online health information; hence, health care professionals should adapt to this shift.

## Introduction

Type 2 diabetes mellitus (T2DM) is a metabolic disorder characterized by chronic hyperglycemia resulting from insulin resistance [1]. By 2025, the International Diabetes Federation estimated that 590 million people worldwide would have diabetes, with approximately 90% of cases attributed to T2DM [2]. Initial management of T2DM typically involves dietary modification and weight loss [3], followed by pharmacological treatment to reduce blood glucose through various mechanisms [4]. Inadequate management increases the risk of complications affecting the cardiovascular, renal, peripheral vascular, ophthalmic, hepatic, or neurological systems [5]. Effective management depends on patient understanding and adherence to therapy, making patient education a fundamental component of T2DM care [6].

The proliferation of digital technologies has led to a marked increase in the number of patients seeking health-related information online [7]. Population-based studies indicate that online health information–seeking behavior rose from approximately 61% in 2008 to 74% in 2017 [7]. In diabetes care, a cross-sectional study in the United States found that 64.5% of 1744 individuals with diabetes engaged in online health information seeking [8]. Digital platforms for accessing health information include websites, search engines, social media, live streaming platforms, and, more recently, artificial intelligence (AI) large language models (LLMs; [9]). This increased reliance on online sources is primarily attributed to their convenience, accessibility, and immediacy [10].

AI LLMs have gained popularity for health information access due to their personalized, empathetic, and conversational interfaces [11]. Research on generative AI for diabetes self-management has demonstrated high patient engagement, attributed to reduced information overload and the delivery of unbiased, transparent, individualized, and evidence-based responses [12]. However, the integration of AI LLMs into health information delivery raises concerns regarding ethical issues, regulatory risks associated with hyperpersonalization, and privacy implications [11,12]. Furthermore, patient safety may be compromised by medical hallucinations, where AI LLMs disseminate misinformation due to inadequate calibration and knowledge gaps [13].

While the quality of traditional online health resources and AI LLM–generated information has been evaluated in previous studies, direct comparisons between these 2 resource types remain limited [14,15]. As patient reliance on AI-generated health content increases, systematic evaluation of its quality relative to established online resources is essential. This study aimed to compare traditional online resources with AI-generated information on T2DM by (1) assessing the overall quality of the resources, (2) evaluating the readability of both information sources, and (3) comparing their reliability and transparency.

## Methods

### Overview

This study used a cross-sectional observational design to evaluate the quality, readability, and transparency of online and AI-generated information related to T2DM. Four predefined search terms were entered into 3 commonly used search engines, and the top 20 search results were retrieved. The 4 search terms were “type 2 diabetes,” “type 2 diabetes mellitus,” “T2DM,” and “adult diabetes.” The search engines used were Google, Yahoo, and Bing. Searches were conducted in Google Chrome using an incognito window, with history and cache cleared prior to the searches. The search was conducted on December 12, 2025. Sponsored results and advertisements were excluded when collating the top 20 search results. The search results were subsequently screened for duplicates and eligibility. In addition to duplicate entries, resources were excluded due to access restrictions (n=7), descriptions of health services rather than educational content (n=2), information about type 1 diabetes mellitus (T1DM; n=1), video-only content (n=2), and textbook purchase links (n=2). Websites presenting information on both T1DM and T2DM were eligible for inclusion, but only material relevant to T2DM was extracted and analyzed. When AI evaluated websites containing information on both T1DM and T2DM, the prompt explicitly instructed it to assess only the T2DM-specific content.

The websites that met our inclusion criteria were classified as public, research-based, hospital-related, or educational (Table 1). Interrater agreement was achieved on the predefined criteria for each category.

**Table 1.** Definitions of website categories.

Category	Definition
Public websites	Consumer-facing health information written for the public using nontechnical language (eg, NHS and Mayo Clinic)
Research-based websites	Sources that publish scientific or academic content, such as peer-reviewed articles, research summaries, or technical reports intended for academic or clinical audiences
Hospital-related websites	Websites produced by hospitals, health care trusts, or clinical institutions, typically containing clinical guidelines, care protocols, or service-specific information
Educational websites	Instructional or textbook-style resources designed for learners (eg, high-school, undergraduate, or early-training health care students) rather than the public

**Figure 1.**
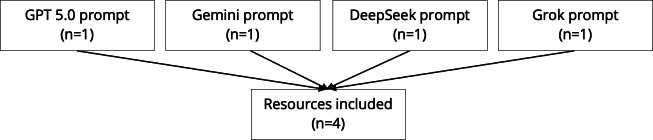
Flowchart displaying the large language models used to generate health information leaflets.

AI-generated leaflets (AIGLs) were created and collated using 4 different AI LLMs: ChatGPT (OpenAI), Gemini (Google LLC), DeepSeek (DeepSeek AI), and Grok (xAI; Figure 1). The same prompt was used across all 4 AI LLMs to create the AIGL: “Create a patient leaflet outlining information regarding type 2 diabetes.” The AI outputs were generated from a single run per AI LLM. Each assessment was conducted in a new chat with all prior history cleared, and a new account was created for each LLM to ensure no previous interactions influenced the results.

The quality of information was assessed using the DISCERN instrument, a valid, evidence-based tool to evaluate the quality of written consumer health information regarding treatment choices [16,17]. The DISCERN instrument consists of 16 items, each scored on a 5-point Likert scale, yielding a maximum score of 80, with higher scores indicating higher quality (Table 2). Each website and AIGL was independently assessed using the DISCERN criteria by 3 independent raters. Additionally, DISCERN scores were generated using ChatGPT to evaluate the same resources, enabling comparison between human and AI LLM assessments. ChatGPT was used to represent AI-rated DISCERN scores as it is the most widely used conversational LLM in health care research, with 92% of all health-related LLM studies using ChatGPT [18]. The prompt provided to ChatGPT stated, “Rate this website [insert website URL] using the DISCERN score. Give it a total score out of 80.” ChatGPT evaluated the full webpage by analyzing the content accessible through the entered URL. When ChatGPT-rated websites containing T1DM and T2DM information, the prompt used was “Rate this website [insert website URL] using the DISCERN score. Give it a total score out of 80. Assess only the information related to type 2 diabetes.”

**Table 2.** DISCERN scores and corresponding quality levels.

DISCERN score	Quality
64‐80	Excellent
52‐63	Good
41‐51	Fair
30‐40	Poor
16‐29	Very poor

Readability was judged using the Flesch-Kincaid Grade Level (FKGL). This metric is widely used in health literacy research to evaluate the accessibility of patient education materials [19]. This measure estimates the educational level required to comprehend a text based on sentence length and word complexity [20]. The score was derived using an online calculator in which the text was entered, and the formula automatically generated the result. The FKGL corresponds to a US school grade level, with lower grade levels indicating greater readability (Table 3).

**Table 3.** Domain categories and corresponding Flesch-Kincaid Grade Level (FKGL), US grade level, and UK educational equivalent.

Descriptive categories	FKGL (US educational stages)	FKGL (UK educational stages)
Very easy	Fifth grade	Year 6
Easy	Sixth grade	Year 7
Fairly easy	Seventh grade	Year 8
Plain English or standard	Eighth and ninth grades	Years 9 and 10
Fairly difficult	10th to 12th grades	Years 11 to 13
Difficult	College	Undergraduate degree student
Very difficult	College graduate	Undergraduate degree graduate

Lastly, the *Journal of the American Medical Association (JAMA)* benchmark criteria were used to explore the transparency and reliability of the resources. The *JAMA* score considers 4 indicators of information reliability: authorship, attribution, disclosure, and currency [21]. Each criterion was recorded as present or absent with a maximum score of 4. The higher the score, the greater its reliability (Table 4).

Normality of continuous variables was assessed using the Shapiro-Wilk test. Both author-rated and AI-rated DISCERN, *JAMA* benchmarks, and FKGL scores demonstrated a *P* value<.05, indicating a significant deviation from normality. The Mann-Whitney *U* test was used to compare scores between websites and AIGLs. The Wilcoxon signed-rank test was conducted to compare author-rated and AI-rated DISCERN scores. Spearman rank correlation coefficient was used to compare variability between author-rated DISCERN scores. Spearman rank correlation was also used to compare ChatGPT-rated and author-rated DISCERN scores for websites. Scatterplots and best-fit lines of DISCERN, *JAMA* benchmarks, and FKGL scores were created based on the top 10 websites’ positions in search engine results. This identified trends between a website’s position and its quality and readability, with slope and *P* value depicting the best-fit line’s significance from nonzero values displayed. A scatterplot with an associated best-fit line was used to graphically represent and identify any association between quality (DISCERN score) and readability (FKGL), with its slope and *P* value reported. Data collection was conducted in Microsoft Excel (16.109.2), and data analysis was conducted on GraphPad Prism (11.0.2; GraphPad Software LLC).

**Table 4.** *Journal of the American Medical Association (JAMA)* scores and corresponding quality levels.

*JAMA* benchmark score	Reliability
0‐1	Poor reliability
2	Moderate reliability
3‐4	High reliability

### Ethical Considerations

An internal research ethics evaluation determined that formal institutional review board, research ethics board, or research ethics committee approval was not required, as the study involved no human participants, patient data, or identifiable information. As no human participants were included, informed consent was not applicable. Privacy and confidentiality were inherently maintained, and no participant compensation was involved.

## Results

### Demographics

**Figure 2.**
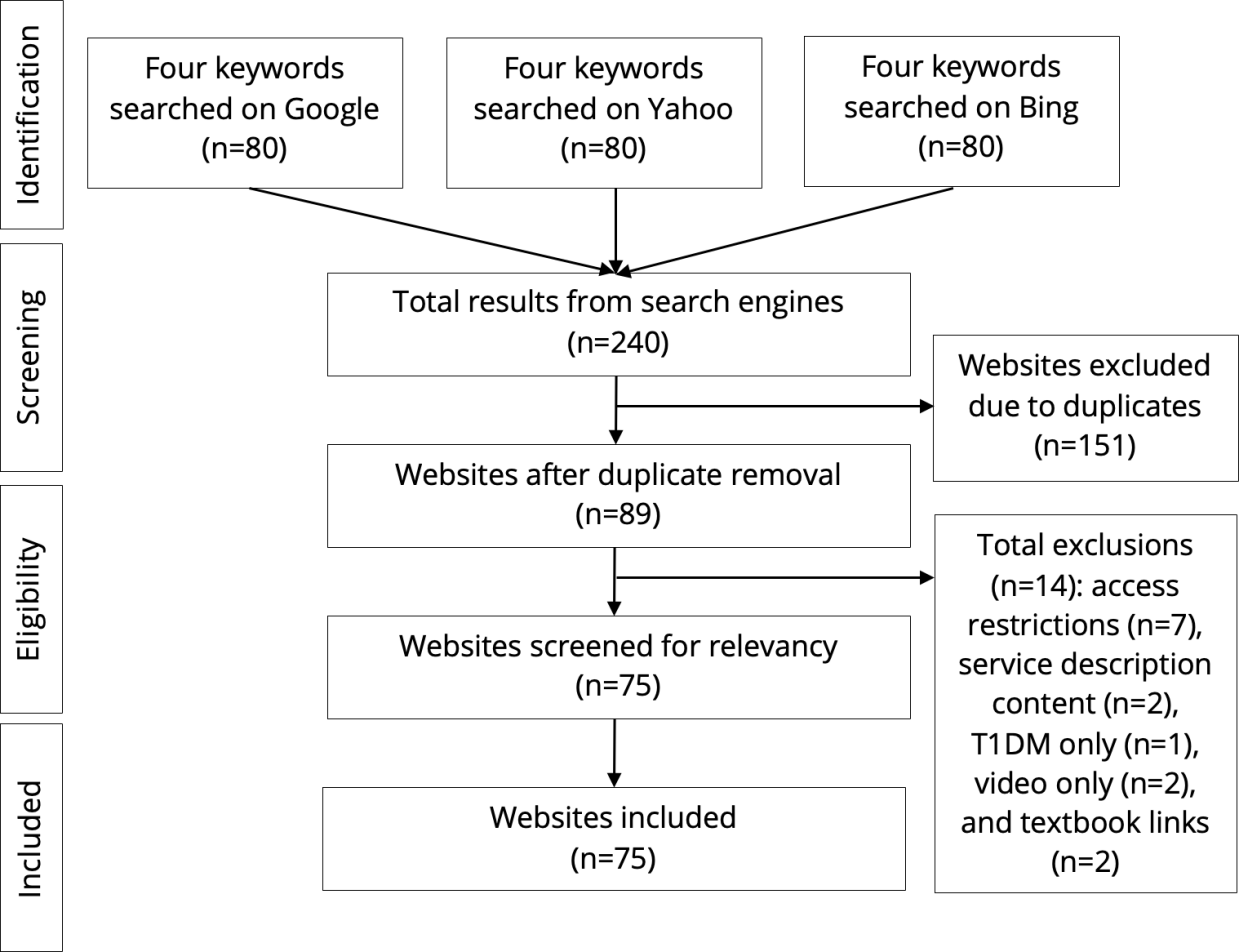
PRISMA (Preferred Reporting Items for Systematic reviews and Meta-Analyses) flowchart illustrating identification, screening, and exclusion of websites. T1DM: type 1 diabetes mellitus.

The study selection process is shown in Figure 2. A total of 75 websites were included in the analysis. Of these, 50 (66.7%) originated in the United Kingdom, 19 (26.3%) in the United States, 5 (6.7%) were of multicountry origin, and 1 (1.3%) was from Spain. Regarding their type, 46 (61.3%) were public information websites, 18 (24%) were research-based, 9 (12%) were hospital-related, 1 (1.3%) was educational, and 1 (1.3%) was from a textbook.

Four AIGLs were produced using ChatGPT, Gemini, DeepSeek, and Grok, and analyzed alongside the websites.

### Quality of Information—DISCERN Scores for Websites vs AIGLs

There was no significant difference in the overall author-rated DISCERN scores between traditional websites and AIGLs (Table 5). Cohen *d* was 0.12 for this, suggesting a minimal effect.

**Table 5.** Comparison of author-rated DISCERN scores between websites and artificial intelligence–generated leaflets (AIGLs).

Source	DISCERN score, mean (SD)	*P* value
Websites (n=75)	42.6 (11.3)	.51
AIGLs (n=4)	43.9 (1.74)	.51

### Quality of Information—Human vs AI DISCERN Ratings

A significant difference (*P*<.001) was found between the author-rated and AI-rated DISCERN scores for websites (n=75), with AI consistently assigning higher quality scores than a human assessor (Table 6).

In contrast, no significant difference was observed between author and AI rankings of AIGLs (n=4) themselves (Table 6).

**Table 6.** Comparison of author-rated and artificial intelligence (AI)–rated DISCERN scores between websites and AI-generated leaflets (AIGLs).

Source	DISCERN score, mean (SD)	*P* value
Websites (n=75)		<.001
Author-rated	42.6 (11.3)	
AI-rated	58.5 (11.5)	
AIGLs (n=4)		.13
Author-rated	43.9 (1.74)	
AI-rated	61.0 (2.94)	

### Quality of Information—AI-Rated DISCERN Comparisons

When assessed exclusively by AI, no significant difference (*P*=.81) was found between the DISCERN scores of websites and AIGL (Table 7).

**Table 7.** Comparison of artificial intelligence (AI)–rated DISCERN scores between websites and AI-generated leaflets (AIGLs).

Source	DISCERN score, mean (SD)	*P* value
Websites (n=75)	58.5 (11.5)	.81
AIGLs (n=4)	61.0 (2.94)	.81

### Reliability and Transparency—*JAMA* Benchmarks

A difference in reliability was identified between the average *JAMA* benchmark scores for websites and AIGLs. Websites achieved substantially higher *JAMA* benchmark scores, while AIGLs scored zero across all criteria (Table 8). The effect size was not calculated as AIGLs scored 0 for the means and standard deviations.

**Table 8.** Comparison of *Journal of the American Medical Association (JAMA)* benchmark scores between websites and artificial intelligence–generated leaflets (AIGLs)^a^.

Source	*JAMA* benchmark score, mean (SD)
Websites (n=75)	2.74 (0.965)
AIGLs (n=4)	0 (0)

a No associated *P* value, as all AIGLs scored 0 on the *JAMA* benchmark.

### Readability—FKGL

No significant difference (*P*=.77) was seen between the FKGL scores of the websites and the AIGLs (Table 9). Both websites and AIGLs required the educational level of an eighth to ninth grader to understand the texts. Cohen *d* was calculated to be 0.13, suggesting a minimal effect.

**Table 9.** Comparison of Flesch-Kincaid Grade Level (FKGL) scores between websites and artificial intelligence–generated leaflets (AIGLs).

Source	FKGl score, mean (SD)	*P* value
Websites (n=75)	8.67 (2.23)	.77
AIGLs (n=4)	8.30 (1.92)	.77

### Associations—Country of Origin

**Figure 3.**
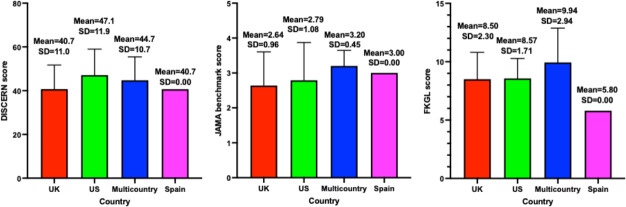
Graphical representation of the means and SDs of DISCERN, *Journal of the American Medical Association (JAMA)* benchmark, and Flesch-Kincaid Grade Level (FKGL) scores based on the origin countries of the websites.

Websites originating from the United States achieved higher quality scores, as indicated by higher DISCERN scores. The highest reliability and transparency, as reflected by the *JAMA* benchmark score, was attained by the “multicountry” category of websites. The most readable websites were also observed in the “multicountry” category (Figure 3).

### Associations—Institutions

**Figure 4.**
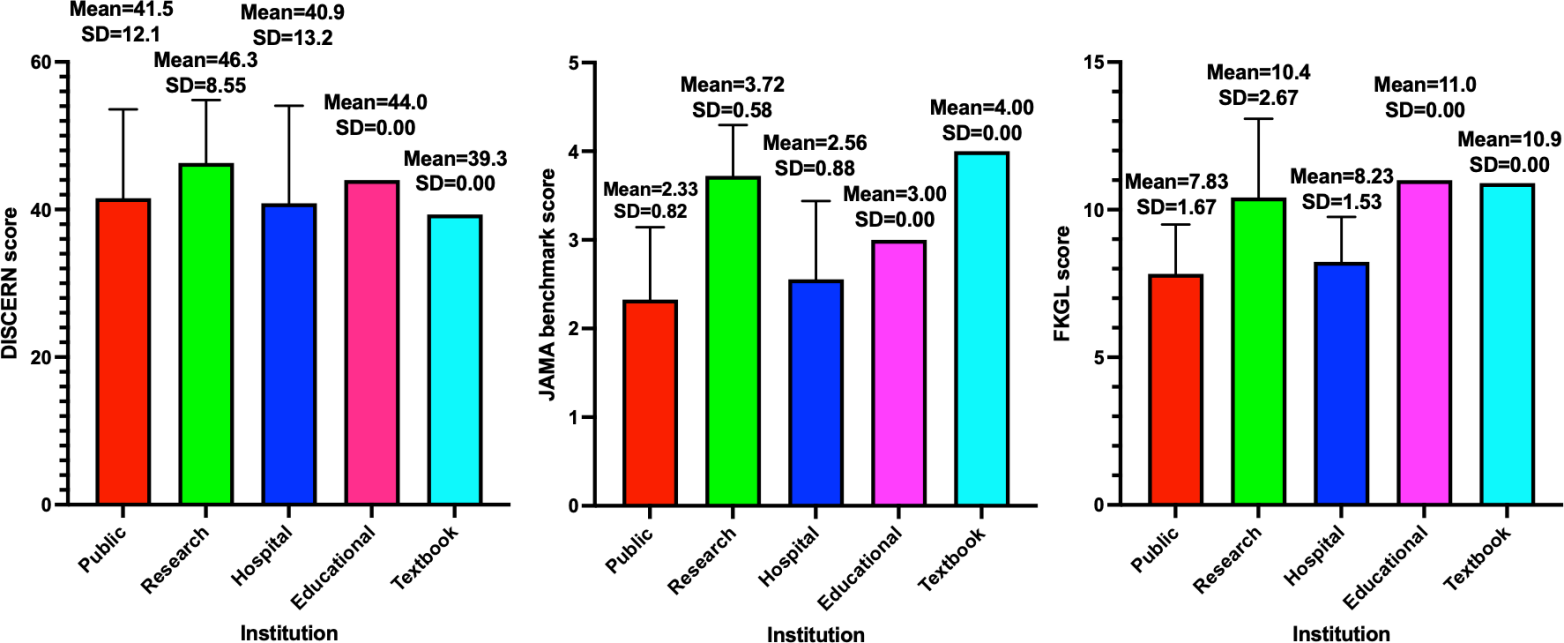
Graphical representation of the means and SDs of DISCERN, *Journal of the American Medical Association (JAMA)* benchmark and Flesch-Kincaid Grade Level (FKGL) scores based on the institutional origin of the website.

Research-based websites (n=18) had the highest DISCERN scores, while the textbook (n=1) had the highest *JAMA* benchmark scores, followed by research articles (n=18). The educational resource website (n=1) and the textbook website (n=1) had the highest FKGL scores. Variability in quality and readability was observed across institutions (Figure 4).

### Search Engine Ranking

**Figure 5.**
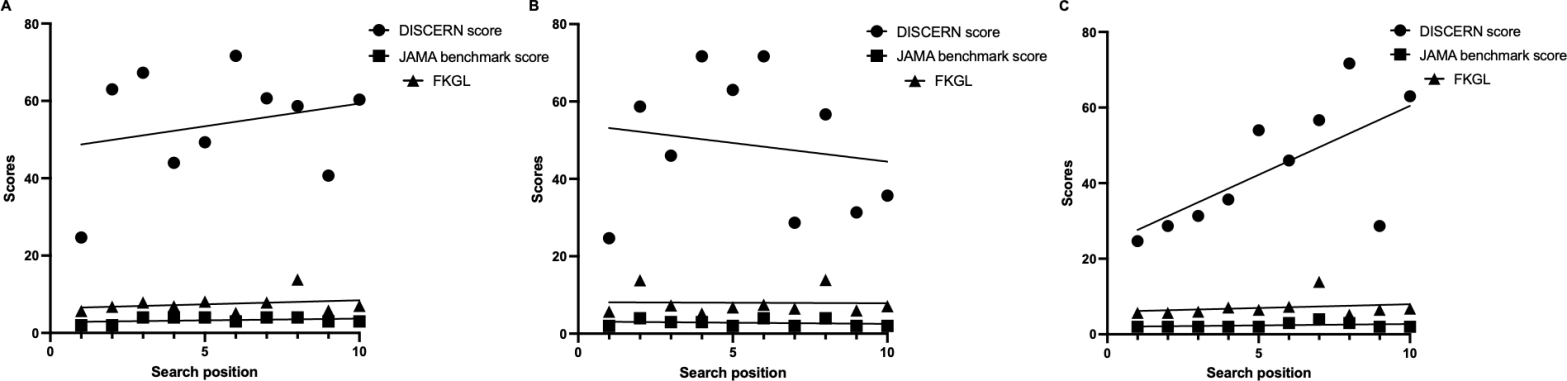
Scatterplots and best-fit lines of DISCERN, *Journal of the American Medical Association (JAMA)* benchmark, and Flesch-Kincaid Grade Level (FKGL) scores for Google, Yahoo, and Bing, based on the websites’ positions in the top 10 search engine results. (A) scatterplot of correlation of DISCERN (slope: +1.175; *P*=.53), *JAMA* benchmark (slope: +0.091; *P* >.99), and FKGL (slope: +0.208; *P*=.59) scores and search position on Google, (B) scatterplot of correlation of DISCERN (slope: −0.996; *P*=.65), *JAMA* benchmark (slope: −0.061; *P*=.58), and FKGL scores (slope: −0.027; *P*=.94) and search position on Yahoo, and (C) scatterplot of correlation of DISCERN (slope: +3.65; *P*=.04), *JAMA* benchmark (slope: +0.073; *P*=.38), and FKGL scores (slope: +0.198; *P*=.50) and search position on Bing. Associated slopes and *P* value representing significance from nonzero slope are represented in tables.

DISCERN, *JAMA* benchmark, and FKGL scores demonstrated a nonsignificant positive trend within the Google search engine. In contrast, all 3 metrics showed a nonsignificant negative trend in the Yahoo search engine. For Bing, the DISCERN score exhibited a significant positive trend, indicating that lower-quality websites tended to appear higher in the search results. The *JAMA* benchmark and FKGL scores, however, showed nonsignificant positive trends in Bing (Figure 5).

### Scoring Correlations

**Figure 6.**
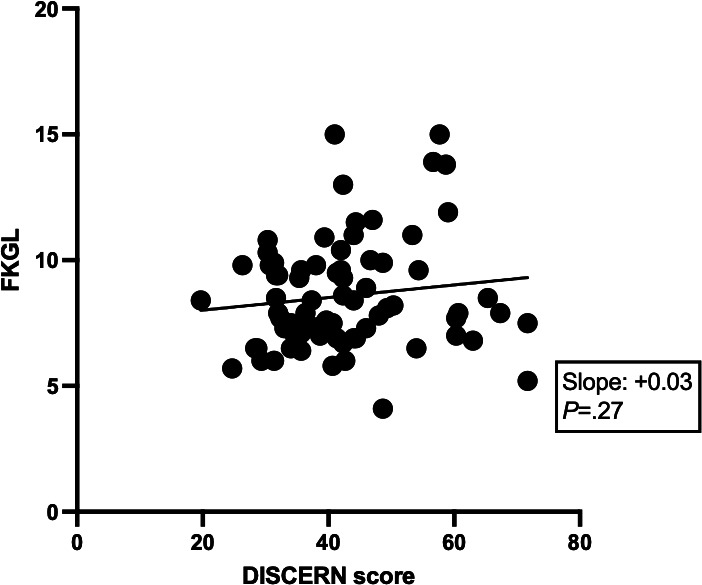
Scatterplot with best-fit line comparing DISCERN scores and Flesch-Kincaid Grade Level (FKGL) with associated slope and *P* value representing significance from nonzero slope.

Comparisons between website quality (DISCERN score) and readability (FKGL) were performed to identify correlations. No significant correlation was found between website quality and readability, suggesting that higher-quality websites were neither more nor less difficult to read (Figure 6).

### Interrater Agreement

There was a strong and significant correlation among the DISCERN score raters, SS, RB, and SM (Table 10). This suggested minimal variability in the DISCERN scoring process between the raters. The sample size for AIGLs (n=4) was too small to generate a valid Spearman rank correlation.

When comparing the author-rated DISCERN scores for websites with those rated by ChatGPT, a notable discrepancy was observed between the 2 raters, indicating that AI applies DISCERN scoring differently than human raters (Table 11).

**Table 10.** Variability assessed using Spearman rank correlation analysis of DISCERN scores between authors SS, RB, and SM.

Source and rater comparison	Spearman *r*	*P* value
Websites		
SS vs RB	0.90	<.0001
SS vs SM	0.88	<.0001
RB vs SM	0.92	<.0001

**Table 11.** Variability calculated by conducting a Spearman rank correlation test between ChatGPT and author-rated DISCERN scores.

Source and rater comparison	Spearman *r*	*P* value
Websites		
ChatGPT vs SS	0.218	.06
ChatGPT vs RB	0.208	.07
ChatGPT vs SM	0.169	.15
Author average vs ChatGPT	0.204	.08

## Discussion

### Principal Findings

Although both online health information and AI have been extensively studied, this is, to our knowledge, the first study to compare them in the context of T2DM.

The overall quality of information provided by websites and AIGLs did not differ significantly when assessed by human evaluators. However, both sources received average author-rated DISCERN scores categorized as “fair quality.” In contrast, ChatGPT assigned both AIGLs and traditional websites an average score categorized as “good quality.” Additionally, the comparison of interrater agreement between authors showed no significant variability according to the Spearman rank correlation, whereas the comparison of DISCERN scores between authors and AI revealed significant categorical variability. This discrepancy raises the possibility that AI may overestimate resource quality by obscuring qualitative differences that human reviewers can identify. Supporting this, a study on the decoy effect found that AI LLMs were more likely to assign higher ratings to misinformation, particularly when false information was more attention-grabbing, whereas humans were better at identifying such inaccuracies [22]. Human assessments, however, also exhibited a decoy effect due to prior knowledge biases [22]. Therefore, while AI LLMs can generate unbiased information, they remain less effective than humans at eliminating errors.

AI-generated content that is factually incorrect yet confidently presented is referred to as a “hallucination” [23]. The phenomenon of hallucinations in newer AI LLMs has been widely discussed [24]. Although recent versions of ChatGPT provide more accurate information, the hallucination rate remains high, at 48%, representing a 15% increase from the previous model [25]. Advancements in AI LLMs have reduced overt errors, making it increasingly difficult for users to distinguish between factual information and confabulations [26]. This challenge is compounded by the assertiveness with which AI LLMs present misinformation. The 6 leading AI LLMs (GPT 4o, Llama-3-70B, Claude 3.5 Sonnet, Gemini, Claude 3 Opus, and GPT 4) demonstrated a mean confidence score of 69.4% when incorrect and 72.5% when correct [27]. Furthermore, AI LLMs have been shown to align with user opinions, even when these are factually inaccurate [28]. The combination of assertiveness and inadequate error elimination poses significant risks to patient safety, as errors may be presented as facts [29].

The rate of AI hallucinations may be reduced if AI LLMs consistently cite their sources. However, all AIGLs produced by the 4 different AI LLMs in this study received a mean *JAMA* benchmark score of 0, indicating a lack of authorship, disclosure, and safeguarding. This finding should be interpreted with caution, as the *JAMA* benchmark was developed to assess traditional web-based health information and may not fully capture the characteristics of AI-generated resources. Consequently, the low scores likely reflect a structural mismatch between the evaluation tool and AI-generated content rather than indicating that the information provided is inherently unreliable. Nevertheless, the absence of these transparency features remains an important limitation because it reduces users’ ability to verify information. In contrast, traditional websites demonstrated greater reliability and transparency, with a mean *JAMA* benchmark score of 2.7. These results are consistent with existing literature, which finds that AI LLMs routinely lack transparency compared with traditional online resources [30]. Additionally, the opaque back end processing, screening, and exclusion processes used by AI LLMs make it difficult for users to assess the trustworthiness of the information provided. In some cases, AI LLMs present broken or irrelevant links and lack diversity in source selection, raising concerns about the underlying algorithms [31].

The development of retrieval-augmented generation offers a promising strategy for reducing the risk of health misinformation [32]. When implemented through either open or closed models integrated with AI LLMs such as ChatGPT, retrieval-augmented generation enables the delivery of up-to-date, dynamically evolving information while continuously verifying outputs against a linked knowledge base [33]. This enables the creation of high-quality, clear information with improved accuracy and personalization, leading to greater patient satisfaction, particularly in the context of T2DM [34,35].

The average reading age in the United Kingdom is 9 years, suggesting that web pages should ideally be written at a fourth- to fifth-grade reading level [36,37]. Both resource types, however, required an eighth- to ninth-grade reading level for comprehension, which exceeds the national average and may hinder access to health information. Furthermore, this study found no significant correlation between DISCERN scores and the readability of resources. Ideally, higher-quality resources should be easier to read and understand to aid accessibility of health information [38]. Given the high prevalence of T2DM in the United Kingdom, efforts to improve patient education are necessary to address health inequalities [39].

The plethora of online health information available through search engines such as Google, Yahoo, and Bing can be overwhelming for users. Therefore, higher-quality websites, as assessed by the DISCERN instrument, would ideally be more prominently ranked in search results to improve accessibility. Google and Yahoo showed a nonsignificant negative correlation between quality and search position, suggesting that website quality was not a significant predictor of search position. Bing demonstrated a significant positive trend with search rank, indicating that websites appearing higher in the search results tended to have lower DISCERN scores. These findings are consistent with previous research indicating that search engine rankings may not correlate with information quality, warranting further investigation into algorithmic differences and their implications for health information access [40,41].

### Limitations

This study should be interpreted within its context. First, although the DISCERN score is a validated and widely used tool for assessing the quality of health information, its application involves a degree of subjective judgment by reviewers. Differences in how individual criteria are interpreted may affect scoring, even when predefined assessment guidelines are used. To minimize this risk, independent assessments were performed by multiple reviewers and interrater reliability was evaluated, demonstrating strong agreement. Nevertheless, the possibility of residual subjective bias cannot be entirely excluded.

Second, the AIGLs were produced using a single standardized prompt across all 4 AI LLMs. Although this ensured uniformity across the leaflets produced, tailoring each AIGL with additional prompts may have yielded higher-quality resources. Additionally, this would replicate users’ patterns of using AI LLMs, as multiple prompts are typically used to elicit information from AI.

This study was restricted to text-based resources because assessing videos and interactive tools posed methodological challenges for AI LLMs. However, most of the population now uses social media posts, videos, infographics, and other interactive formats when obtaining health information [42,43]. As content sources vary substantially in quality, readability, and reliability, the findings may not fully reflect the broader digital health information landscape.

The discrepancy in sample sizes between websites (n=75) and AIGL (n=4) remains a limitation due to differences in statistical power. The lack of statistically significant differences observed in this study should not be interpreted as evidence of equivalence between the groups. Given the relatively small sample size, the study may have been underpowered to detect modest but potentially meaningful differences in website quality and reliability metrics. Consequently, the absence of statistical significance may reflect insufficient statistical power rather than a true lack of difference. Future studies incorporating larger sample sizes and a priori power calculations are warranted to confirm these findings and better characterize any underlying differences. This limitation should be considered when interpreting the results and their generalizability.

Finally, given that this study used a cross-sectional design, it does not capture the dynamic nature of websites and AI LLMs. Future studies should be longitudinal to incorporate changes in quality and reliability over time, as these resources are constantly updated and revised.

### Conclusions

This study demonstrated that both traditional websites and AI-generated resources offer T2DM information of comparable quality; however, the overall standard remains suboptimal considering the global prevalence and clinical burden of the condition. Additionally, readability levels exceeded recommended thresholds for patient education, raising concerns about accessibility and the potential to exacerbate health inequalities.

Although AI-generated content achieved quality scores similar to those of traditional websites, it did not meet basic reliability criteria as assessed by the JAMA benchmark. The absence of safeguarding measures raises major concerns related to patient safety and governance.

The landscape of medical encounters is evolving, with patients increasingly presenting with preconceived notions based on online health information. Health care professionals should be mindful of this shift during consultations. Prioritizing improvements in the quality, readability, and safeguards of online health information is essential.

## Abbreviations

AI: artificial intelligence
AI LLM: artificial intelligence large language model
AIGL: artificial intelligence–generated leaflet
FKGL: Flesch-Kincaid Grade Level
JAMA: Journal of the American Medical Association
T1DM: type 1 diabetes mellitus
T2DM: type 2 diabetes mellitus
